# Coping Strategies for Landslide and Flood Disasters: A Qualitative Study of Mt. Elgon Region, Uganda

**DOI:** 10.1371/currents.dis.4250a225860babf3601a18e33e172d8b

**Published:** 2016-07-11

**Authors:** Jimmy Osuret, Lynn M Atuyambe, Roy William Mayega, Julius Ssentongo, Nathan Tumuhamye, Grace Mongo Bua, Doreen Tuhebwe, William Bazeyo

**Affiliations:** School of Public Health, Department of Disease Control and Environmental Health, College of Health Sciences, Makerere University, Kampala, Uganda; Department of Community Health and Behavioural Sciences, School of Public Health, College of Health Sciences, Makerere University, Kampala, Uganda; Department of Epidemiology and Biostatistics, School of Public Health, College of Health Sciences, Makerere University, Kampala, Uganda; School of Public Health-Resilient Africa Network, College of Health Sciences, Makerere University, Kampala, Uganda; Department of Epidemiology and Biostatistics, School of Public Health-Resilient Africa Network, College of Health Sciences, Makerere University, Kampala, Uganda; School of Public Health-Resilient Africa Network, College of Health Sciences, Makerere University, Kampala, Uganda; Department of Health Policy, Planning & Management, School of Public Health, College of Health Sciences, Makerere University, Kampala, Uganda; Department of Disease Control and Environmental Health, School of Public Health, College of Health Sciences, Makerere University, Kampala, Uganda

**Keywords:** coping, floods, landslides, uganda

## Abstract

Introduction: The occurrence of landslides and floods in East Africa has increased over the past decades with enormous Public Health implications and massive alterations in the lives of those affected. In Uganda, the Elgon region is reported to have the highest occurrence of landslides and floods making this area vulnerable. This study aimed at understanding both coping strategies and the underlying causes of vulnerability to landslides and floods in the Mt. Elgon region.

Methods: We conducted a qualitative study in three districts of Bududa, Manafwa and Butalejja in the Mt. Elgon region in eastern Uganda. Six Focus Group Discussions (FGDs) and eight Key Informant Interviews (KIIs) were conducted. We used trained research assistants (moderator and note taker) to collect data. All discussions were audio taped, and were transcribed verbatim before analysis. We explored both coping strategies and underlying causes of vulnerability. Data were analysed using latent content analysis; through identifying codes from which basis categories were generated and grouped into themes.

Results: The positive coping strategies used to deal with landslides and floods included adoption of good farming methods, support from government and other partners, livelihood diversification and using indigenous knowledge in weather forecasting and preparedness. Relocation was identified as unsustainable because people often returned back to high risk areas. The key underlying causes of vulnerability were; poverty, population pressure making people move to high risk areas, unsatisfactory knowledge on disaster preparedness and, cultural beliefs affecting people’s ability to cope.

Conclusion: This study revealed that deep rooted links to poverty, culture and unsatisfactory knowledge on disaster preparedness were responsible for failure to overcome the effects to landslides and floods in disaster prone communities of Uganda. However, good farming practices and support from the government and implementation partners were shown to be effective in enabling the community to lessen the negative effects disasters. This calls for high impact innovative interventions focused in addressing these underlying causes as well as involvement of all stakeholders in scaling the effective coping strategies in order to build resilience in this community and other similarly affected areas.

Key words: Coping, Underlying causes, Floods, Landslides, Mt. Elgon, Uganda

## Introduction

Landslides and floods are one of the most important disasters today with floods alone reported to account for 6.8 million deaths worldwide 1 . Estimates indicate that Asia and Africa are among the most vulnerable regions prone to disasters with Asia alone accounting for about 50% of flood related death^1^^,^ 2. The occurrence of landslides and floods in East Africa has increased over the past decades with enormous Public Health implications and massive alterations in the lives of those affected^3^. Uganda is one of the African countries most prone to disasters. In 2010, flooding of the banks of river Manafwa and landslides in Bududa district in the Mt. Elgon region left 5,000 individuals displaced and over 400 killed^4^. The Mt. Elgon region of Uganda is reported to have the highest rates of landslides and floods in the country 3with devastating effects on the livelihood of people. The key primary effects to landslides and floods in the Mt. Elgon region include loss of life and injuries, destruction of infrastructure, destruction of farm land and livestock and destruction of property and business 4 ^,^ 5. In the long run, communities with broken sanitation facilities, disrupted education systems, malnutrition and poverty are susceptible to secondary effects such as famine, disease outbreak 4 ^,^ 6.

Previous studies have also reported a number of factors predisposing people, infrastructure and institutions to the effects of landslides and floods among which include; settling in high risk areas such as mountain slopes, lack of information on mitigation measures to reduce the effects of landslides; instability of slopes; the informal nature of houses which makes them prone to collapsing in the event of a landslide; and low level of preparedness in the district^5^. In the event of a disaster, social characteristics of household members such as age, sex, health status and disability increase vulnerability to the disaster effects^7^. Oftentimes, women, children, the sick and the elderly have been reported to be the most at risk groups affected by landslides and floods. In particular the young children and elderly are vulnerable because they are too weak to run and often times remain at home and miss out on the warnings about the threat of landslides and floods^5^.

In sub-Saharan countries particularly, Uganda, Ethiopia and Rwanda, individuals, households and communities have come up with some local coping strategies.Coping strategies are a combination of all the strengths, attributes and resources available within a community, society or organization that can be used to avert some or all of the negative effects of a shock or stress^3^. For instance, relocation to safer areas if the threats are too great to ignore, receipt of aid and relief, and resorting to subsistence and innovative farming practices such as terracing in order to overcome crop destruction following heavy rains^3^^,^ 8. In Uganda the national policy for disaster preparedness and management addresses some key coping issues such as resettlement of people living in high risk areas, applying appropriate farming technologies and prohibition of settlement in high risk areas. However, implementation of the Policy actions on landslides remains significantly inadequate. This could probably be because of the limited capacities to manage and reduce disaster risks at both community and national level 9.

In the current advent of climate change and the changing environment, it is anticipated that landslide and flood incidents will be on the increase within exposed communities in Mt Elgon region. However, there is still limited qualitative information on the coping strategies and underlying causes of vulnerability to the effects of landslides and floods in the Mt. Elgon region. This study explored underlying causes and coping strategies used to avert the effects of landslides and floods in the Mt. Elgon region. Therefore, findings on the strategies that have been used to mitigate, to cope positively, recover and learn from the landslides and floods could inform the design of appropriate innovative solutions that can strengthen the capacity of affected populations to become resilient.

## Methods

**Study setting**

This study was conducted in three districts of Bududa, Manafwa, Butalejja located in the Mt. Elgon region in eastern Uganda. The Elgon region has semi urban and rural communities with an estimated population of 1,795,567. The population is engaged in various economic and social activities including; subsistence farming of both cash and food crops, business/trade, rearing domestic animals like goats among others^5^.

**Study design**

We conducted a qualitative study that used Focus Group Discussions (FGDs) and Key Informant Interviews (KIIs). FGDs and KIIs were best suited to explore underlying causes of vulnerability and the coping strategies for landslides and floods.

**Selection of study participants**

A total of 48 participants were involved in the 6 FGDs. The FGDs were drawn from the community members, mainly opinion leaders, political leaders, cultural leaders and other categories of individuals who had a good knowledge of the development challenges of the specific community. All participants were aged 18 years and above. Participants from each focus group had both males and females. Given that gender did not influence behaviour as regards disasters, both males and females were interviewed in the same focus group^3^. FGDs participants were from rural areas worst affected by floods and landslides. We also conducted 8 KIIs with District Technical Officials, representatives from NGOs and district Political/ Opinion/Religious leaders from Bududa, Manafwa and Butalejja.

**Data collection**

The focus of the study was resilience which dealt with the capacity of people and systems to mitigate, cope positively, recover and learn from shocks and stresses in a manner that reduces vulnerability and increases well-being^3^. The target districts for this study were Bududa, Manafwa and Butalejja – the most affected communities in the region. We then randomly selected two high risk sub counties from each of the districts ensuring inclusion of rural localities. In each sub county, we conducted two FGDs.

We recruited five experienced research assistants who also had a good working knowledge of English, and the local languages (Lugishu-luomasaba for Bududa/Manafwa and Lunyole for Butaleja, and trained them on the study protocol and procedures. The survey questionnaires were translated into the local language and pretested in a similar setting in order to get feedback on questions that were not clear. This was done prior to data collection. Investigators also participated in the data collection process.

During interviews we asked open ended questions followed by targeted questions on predetermined categories. The interview guide focused on factors that empower communities to resist disasters and underlying factors that make communities fail to overcome their vulnerabilities (probing for people, physical infrastructure, livelihoods infrastructure and institutions). The FGDs and KIIs were audio recorded with consent.

**Data management and analysis**

The FGDs and KIIs were all transcribed verbatim and those in the local languages translated without altering the meaning. A conventional content analysis approach was used as described by Hsiu-Fang and Sarah^10^ with codes and categories arising from the data. Analysis was done in two stages, first, the manifest content analysis and then the latent content analysis. The transcripts were read and re-read by the authors to achieve immersion. Text data was read to derive codes by highlighting emerging factors based on our understanding of the data. Codes were sorted into categories based on their linkages. The categories were grouped together into meaningful overarching themes i.e. coping strategies and underlying causes of vulnerability to the effects of landslides and floods. Strategies that improved long term vulnerability of individuals, households and communities were categorised as positive coping while those strategies that had no impact were put under the category of unsustainable coping^3^.

**Ethics Statement**

Ethical clearance was obtained from Makerere University School of Public Health Research and Ethics Committee (IRB00011353) and Uganda National Council for Science and Technology (SS3357). The objectives, benefits and risks of the study were explained to the study participants and informed consent obtained. All data obtained during the study were treated with confidentiality and anonymity. We restricted data access to only the investigators and the research assistants.

## Results

The results are presented in two thematic topics from the data analysis namely; coping strategies and underlying causes of vulnerability to landslides and floods as discussed in the FGDs and KIIs. A summary of the key results are presented in Table 1.

**Figure:**
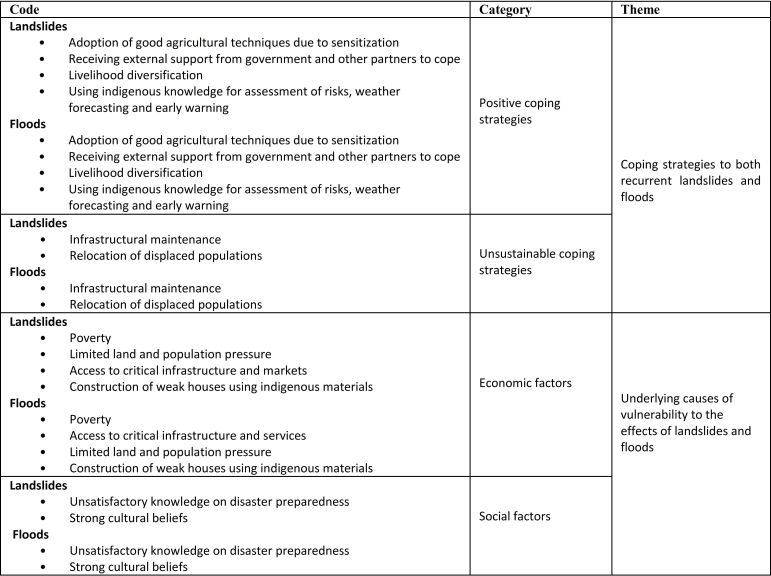
**Table 1.** **Summary of Results: Emerging Codes**

**Coping strategies for recurrent landslides and floods in the Elgon region**

Disasters have occurred repeatedly in the Mt. Elgon region. Focus Group discussions and key informants described a number of coping strategies that have been used by individuals and communities to lessen the effects of landslides and floods. Five FGDs and half of key informants reported adoption of good agricultural techniques e.g. tree planting and terracing as a positive coping strategy for reducing the effects of floods and landslides. This was as a result of sensitization and public awareness of the community on disaster mitigation measures. Majority of the key informants explained that sensitization and training were conducted by various agencies to help cope with the effects of the disaster.

*We have got NAADs coordinators and service providers who are training people in land soil conservation and better farming practices. We also had NGOs supporting communities in tree planting. The district natural resource office has been able to establish nursery trees where people are given free tree seeds to plant in their gardens*(Key Informant technical officer, Manafwa)

Communities relied on external support from government and other partners in coping with floods and landslides. Majority of key informants and three FGDs mentioned support from government and other partners e.g. in putting up of flood embankments, terracing, tree planting, digging trenches as important in coping with landslides and floods.

*The government is also there with National Environment Management Authority (NEMA) and the district environmental office with some interventions e.g. Tree planting, zero grazing, terracing, contour ploughing.*(Key Informant representative from NGO, Bududa)

Communities reported small scale diversified wealth creation activities to improve household incomes including small scale businesses on major trading centres, agricultural diversification like poultry and livestock farming and seeking formal employment.

*Some of us have established retail shops at Bulucheke Trading centre, so when our crops are destroyed by the landslides, we survive on income from these shops. We also acquire some money to pay for school fees for our children and when they finish and get employed, they support us especially in the event of a landslide *(FGD, Bulucheke Bududa District)

As a positive coping strategy, one key informant mentioned that local communities relied on traditional indigenous knowledge systems for recognition of landslides and floods especially in assessment of risks, weather forecasting and early warning. They use signs to tell whether it is going to rain heavily and experience a landslide or flood such as the appearance of cracks on the lands.

*Some of the people see cracks and move after seeing cracks. But mainly after they have seen cracks they report to the district that look here there is this problem.* (Key Informant District technical officer, Bududa)

However, from a resilience perspective, some of the coping strategies used in managing landslides and floods were ineffective in lessening the effects of these disasters hence unsustainable^3^. These included infrastructural maintenance and relocation of displaced populations. Nearly all of the FGDs mentioned infrastructure maintenance such as community road maintenance, creation of water channels, digging trenches and sealing river banks to be unsustainable in reducing the effects of floods and landslides. They explained that these disasters still come and destroy everything on their way.

*We have tried to dig trenches but it has failed because when the waters come, the area still floods. Even when we try to raise up the roads, the roads still flood.* (FGD, Butalejja)

Although two thirds of the FGDs mentioned relocation as a positive coping strategy, Three key informants were concerned with its effectiveness pointing out that some of the relocated people often returned back to high risk areas due some factors like cultural attachments, funding challenges and failure to involve and consult the community in planning.

*One time government tried to shift people from vulnerable places to Kiryandongo, but they never stayed there long. They came back because of their traditional beliefs. *(Key Informant Key informant, politician Manafwa)

*They didn’t consult people and they did not look at what people thought was okay for them. They thought for them, they planned for them, they decided for them they took them to Kiryandongo (relocated) and they were not prepared. Even if it was me I would not stay there. *(Key Informant District technical officer, Bududa)

**Underlying causes of vulnerability to landslides and floods**

Many of the factors which exacerbate vulnerability to the effects of landslides and floods in the Mt. Elgon region are mainly socio-economic and cultural. The main economic causes that emerged from the FGDs and key informants were, poverty, construction of weak houses using indigenous materials, limited land, population pressure and lack of access to critical infrastructure and services. Consistently across all FGDs and key Informants, the issue of poverty was highlighted as the main cause of vulnerability for landslides and floods. For example it was pointed out that some communities did not have resources to come up with simple measures or innovations to reduce the effects of landslides and floods. Poverty was linked to the non-durable informal structures of houses that were being built. Five of the key informants mentioned that the houses were built using poor quality materials and therefore could not resist floods or landslides.

*They (community) are powerless because they are poor, they cannot afford to put in place technologies, they don’t even have labour, you don’t have the resources because labour is expensive so that alone (poverty) is the biggest hindrance.* (Key informant District technical officer, Butalejja)

*The materials they used are not good, in other words the contractors do shoddy work when the river floods, automatically the water will come and sweep away the structures because of the weak foundation.* (Key Informant representative from NGO, Manafwa)

Half of the FGDs mentioned population pressure plus land shortage to force people live in high risk areas. Population pressure could also influence negative land use patterns e.g. deforestation. There was also a widely held view among two key informants that inadequate services and difficulty in accessing critical infrastructure such as roads hindered communities from accessing markets for their produce leading to income insecurity. This complicates survival efforts

*We are constructing in places that we are not supposed to because we are over populated and have nowhere to go .We end up constructing in swamps and wetlands that are prone to floods.* (FGD Manafwa Wesswa)

*Because communication lines have been cut, roads are not working, people are not accessing the sellers (because of landslides and floods) and in other words these people are affected in the nature that they don’t sell their produce in a time or at a favourable prices and their income become very low( their income are affected.* (Key informant representative from NGO, Bududa)

The main social causes of vulnerability that emerged were unsatisfactory knowledge on disaster preparedness and strong cultural beliefs among community members. Half of the FGDs mentioned limited knowledge about disaster preparedness and mitigation in increasing the community’s vulnerability to landslides and floods. In addition two FGDs mentioned the influence of strong traditional beliefs in making people fail to overcome their vulnerability e.g. settling in high risk areas reported to be their ancestral homes.

*We are primitive and do not know what to do to mitigate these problems. This problem can also be attributed to the fact that our children are lowly educated. *(FGD Manafwa Bukokho)

*This can also be attributed to the cultural beliefs, for example, I know of some old man that said that his grand parents and parents were buried here and so he was going no where else but rather stay in his ancestral lands.* *(FGD Bududa Bulecheke)*

## Discussion

The study findings clearly show that some strategies that were used to cope with the effects of landslides and floods had a positive impact and improved wellbeing of communities such as adoption of good farming methods, support from government and other partners, livelihood diversification and using indigenous knowledge in weather forecasting. However, some strategies used in coping were unsustainable and had not built permanent protection from recurrent landslides and floods such as infrastructural maintenance and relocation of displaced populations.

Coping strategies employed by farmers to reduce the impact of landslides and floods in the Elgon region included soil conservation practices and diversification with tree planting, contour farming, and terracing. These practices have been reported to be fairly effective in lessening the effects of shallow landslides and run off from floods 11 and should be emphasized. Uptake of sustainable farming was as a result of community sensitization on mitigation measures. Generally, education programmes about disasters mitigation have been reported to increase hazard knowledge and this is associated with increase in disaster preparedness behavior 12. This is because people who are sensitized and aware of hazards in their communities are more likely to perceive themselves to be at risk. Communities that perceive themselves to be at risk are more likely to prepare and mitigate future hazardous events thus promoting community resilience 13. Therefore disaster awareness creation at individual, community and organizational level could be a more effective tool in mitigating of disaster risk and ameliorating their effects.

The study also showed that external support from government and other partners in coping with landslides and floods was an effective strategy in helping communities to lessen the effects of landslides and floods. The government of Uganda together with humanitarian agencies have been taking action to reduce the effects of disasters^14^^,^ 15. Some of the activities carried out by government and partners included; early warning and training activities designed to enhance preparedness^3^. However, it must be noted that in dealing with disasters the Government of Uganda has shifted its paradigm from response orientated to preparedness and mitigation as one of the effective strategies in overcoming the increasing negative effects of hazards that accompany population growth, development and climate change 17.

This paper identified some positive coping attempts used by individuals and households to raise income through livelihood diversification e.g. setting up small retail shops and agricultural diversification with poultry and livestock farming. One of the motivations for undertaking diversification opportunities was that households could continue to survive on the income generated in the event that landslides and floods destroyed their crops thus lessening the effects of the shocks and stresses. Similar coping strategies employing crop diversification, and livelihood diversification have been employed among rural communities in sub Saharan Africa because of environmental uncertainty related to climate variability that makes farming risky^18^. This has implications for the local authority to support local livelihood initiatives by communities since it could address some underlying causes of vulnerability such as poverty.

The pattern in data indicated that indigenous knowledge was used for assessment of risks, vulnerability, weather forecasting and early warning. Similar coping strategies utilizing traditional indigenous knowledge to facilitate understanding of disaster phenomenon have also been reported among other communities in sub-Saharan Africa and low middle income countries (LMICs)^19^^,^ 20. Indigenous knowledge about the weather patterns can promote disaster preparedness and prevention. Some studies in the Mt. Elgon region and elsewhere have indicated that the understanding of landslides and other disasters in the community was from a religious, local and cultural perspective^21^ with assertions that the disasters could be predicted using indicators like the position of the sun, height of bird nests near rivers^20^. Indigenous Knowledge has evolved over time and is based on observations and experiences passed on through many generations^20^. Such uninformed trends coupled with the increasing variability in climate make it difficult to anticipate where and when an event might occur making disaster management and planning at community level difficult 22. Thus the need to identify local knowledge and practices about landslides and floods that can be integrated with modern science. This could inform decision making by communities, practitioners, policy makers and other stakeholders.

However, our results also show that some of the strategies that have been used to cope with landslides and floods were ineffective and did not build permanent protection from the effects of landslides and floods such as relocation of displaced populations. This was not sustainable in the long term as people often returned to the high risk areas. The possible explanation for this could partly be due to strong cultural ties/attachment to the land, fear that their land was going to be grabbed and the high costs associated with moving^3^^,^ 21. Much as relocation appeared to be unsustainable in Ethiopia and Uganda, in Somalia it was reported to be a positive coping, as the existential threats in some areas were simply too great to ignore^3^.

The key underlying causes of vulnerability included; poverty, limited land and population pressure making people move to high risk areas, access to critical infrastructure, construction of houses with weak indigenous materials, lack of awareness on preventive measures, cultural beliefs affecting people’s ability to cope and lack of political will.

The study revealed poverty to be one of the factors promoting vulnerability to landslides and floods and this is similar to that reported in studies conducted in Uganda and other developing countries that showed a link between poverty and risk to vulnerability to disasters 23 ^,^ 24 ^,^ 25. The possible explanation for the correlation between poverty and higher risk could partly be due to insufficient capacity by poor communities to prepare to cope with disasters thus increasing their vulnerability. Low income communities have been shown to suffer the highest risk of disasters because they live in poor quality housing and in informal settlements that are prone to floods and landslides. This is because they are powerless cannot afford measures to lessen the effects of disasters 25. This therefore requires a multidisciplinary approach in incubating high impact innovations to address the effects of these disasters.

Lack of basic infrastructure like roads, telecommunication, health and education facilities or markets to sell their produce emerged as part of the underlying causes of vulnerability to the effects of landslides and floods in the Mt. Elgon region. This problem has been reported in other countries like Malawi where communities lack infrastructure and services to withstand the effects floods^3^. Therefore, interventions that support flood and landslide affected communities to access and have leverage in the market should be promoted. In addition, priorities should be to build physical infrastructure, increase access to formal education, and decrease dependence on agriculture through diversification.

The communities identified practices like deforestation and over cultivation as a result of increasing population pressure to have greatly contributed to landslides and floods. Deforestation and excavation of slopes for house construction has been reported to reduce the stability of soils in mountainous areas making it prone to mudslides^26^. These findings are consistent with those in studies conducted in Mt. Elgon slopes and other disaster prone areas that reported a correlation between population increase and negative land use practices^21^. Rapid population increase among poor communities has been reported to increase the demand for food and wood fuel thus resulting into environmental degradation that also threatens food production. High population growth increases the pressure on land and this triggers exposure to disaster risk hence increasing vulnerability^27^. Population pressure could drive people to settle in flood prone and disaster prone areas. This has implications for mapping disaster prone areas that are not suitable for settlement.

The study also revealed unsatisfactory knowledge on disaster preparedness and mitigation to be one of the factors promoting vulnerability to landslides and floods and this is similar to that reported in studies conducted in Uganda and other developing countries^23^. Understanding weather and climate patterns is essential in enabling communities make decisions and develop solutions to climate related shocks and stresses^28^. However, most poor countries do not carry out awareness raising and risk communication and this delays evacuations making communities more vulnerable to disasters^29^.

The world disaster report corroborates our findings on the influence of strong traditional beliefs in making people fail to overcome their vulnerability. Many communities around the world have a cultural attachment and perception to disasters that affect how people prioritize risk thus making them vulnerable^30^. The fact that people settle in high risk areas with assertions that ‘those are their ancestral homes’ increases their vulnerability and eventually undesired outcomes in case disasters occur^31^. These situations make disaster prevention difficult. These findings have implications for disaster risk reduction programmes to take into account and incorporate people’s cultures, beliefs and attitudes in order to increase the community’s willingness to support any interventions.

## Conclusion

This study reveals that failure to overcome the effects to landslides and floods in disaster prone communities of Uganda are mainly due to deep rooted links to poverty, culture and unsatisfactory knowledge. Good farming practices and support from the government and implementation partners were shown to be effective in enabling the community to resist the effects disasters. This calls for support in designing more focused interventions targeting reduction of these underlying factors as well as involvement of all stakeholders in scaling the effective coping strategies in order to build resilience in this community and other similarly affected areas.

## Conflict of interest

The authors have declared that no conflicts of interest exist.
